# Sex and age differences in the use of medications for diabetes and cardiovascular risk factors among 25,733 people with diabetes

**DOI:** 10.1371/journal.pone.0287599

**Published:** 2023-10-24

**Authors:** Crystal M. Y. Lee, Alice A. Gibson, Jacob Humphries, Natasha Nassar, Stephen Colagiuri

**Affiliations:** 1 Faculty of Medicine and Health, University of Sydney, Sydney, New South Wales, Australia; 2 School of Population Health, Curtin University, Perth, Western Australia, Australia; 3 Menzies Centre for Health Policy and Economics, School of Public Health, Faculty of Medicine and Health, University of Sydney, Sydney, New South Wales, Australia; 4 Charles Perkins Centre, University of Sydney, Sydney, New South Wales, Australia; Karolinska Institutet, SWEDEN

## Abstract

**Aim:**

To determine sex and age differences in the use of medications for diabetes and cardiovascular risk factors in people with diabetes in Australia.

**Methods:**

Pharmaceutical claims data of participants in the 45 and Up Study who self-reported having diabetes before 2013, were alive on 1^st^ January 2013 and had at least one medication dispensing record between 1^st^ January 2013 and 31^st^ December 2019 were analysed. Annual sex and age-specific percentages of participants supplied specific medications were estimated for years 2013 to 2019. Percentages were reported for any glucose lowering medications and by drug class, any lipid modifying agents, and any blood pressure lowering medications.

**Results:**

Altogether 25,733 participants (45.2% women) with diabetes were included. The percentage of participants who were supplied with glucose lowering medications was consistently lower in women compared to men. In both sexes, the percentage of participants who were supplied with glucose lowering medications was lowest among those aged ≥75 years and this decreased over time. Similar findings were observed for lipid modifying agents and blood pressure lowering medications. The use of sodium glucose co-transporter 2 inhibitors increased substantially in participants aged <75 years since it became available in 2013. However, no sex differences were observed in its use among people with hospital-recorded history of cardiovascular disease.

**Conclusions:**

Practitioners should be aware of possible sex disparities in the pharmacological treatment of diabetes and cardiovascular risk factors in people with diabetes in Australia. There is a possible time lag between reporting of research findings and uptake of sodium glucose co-transporter 2 inhibitors prescribing in individuals with diabetes and high cardiovascular risk in clinical practice, nevertheless, the result observed was consistent with the management guidelines at the time of the study.

## Introduction

Women with diabetes are at greater excess risk of coronary heart disease and stroke than men with diabetes [1, 2]. Under-prescribing of cardiovascular medications in women with cardiovascular disease compared to men with cardiovascular disease has been reported in Australia and elsewhere [3, 4]. Sex disparities in the pharmacological treatment of people with diabetes is less definitive [5–7].

There are an estimated 1.2 million individuals with diabetes in Australia, 88% of whom are aged ≥45 years [8]. The Australian population receiving glucose lowering medications through the government subsidised Pharmaceutical Benefits Scheme (PBS) and Repatriation PBS (RPBS) has increased from 811,009 individuals in 2012 to 928,561 individuals in 2016, which cost $514 million in 2015–16 [9]. Despite the increasing demand for glucose lowering medications, the last national survey with a biomedical component reported only 55% of women and men aged ≥18 years with known diabetes achieved the recommended glycated haemoglobin (HbA1c) treatment target in 2011–12 [10]. A patient experience survey in 2018–19 reported 6.7% of individuals receiving a prescription for any medication in the past 12 months delayed getting or did not get the prescribed medication due to cost, and delays were more prevalent in people who were living in the most socioeconomically disadvantaged areas compared to those living in the least disadvantaged areas and in women compared to men [11]. While most prescription medications are heavily subsidised by the Government, each subsidised medication could still cost up to $42.5 for general patients and $6.8 for concession card holders (2022 figures) [12].

Previous reports on medication use in people with diabetes in Australia have used self-reported survey data and incomplete PBS claims data that did not include prescriptions priced under the general patient co-payment prior to mid-2012 (i.e. medications such as metformin, which cost less than the maximum patient medication cost set by the government) and/or only included PBS claims data of concession card holders [13–15]. Another report used the full PBS dataset [9] but did not include socio-demographic indicators and medical history, which are not collected in the PBS dataset. Understanding medication use in people with diabetes is an important step to achieving optimal health outcomes. We, therefore, aimed to describe the use of glucose lowering and cardiovascular medications among middle-aged and older people with diabetes in Australia and explore sex and age differences.

## Methods

### Study participants

**Fig 1:**
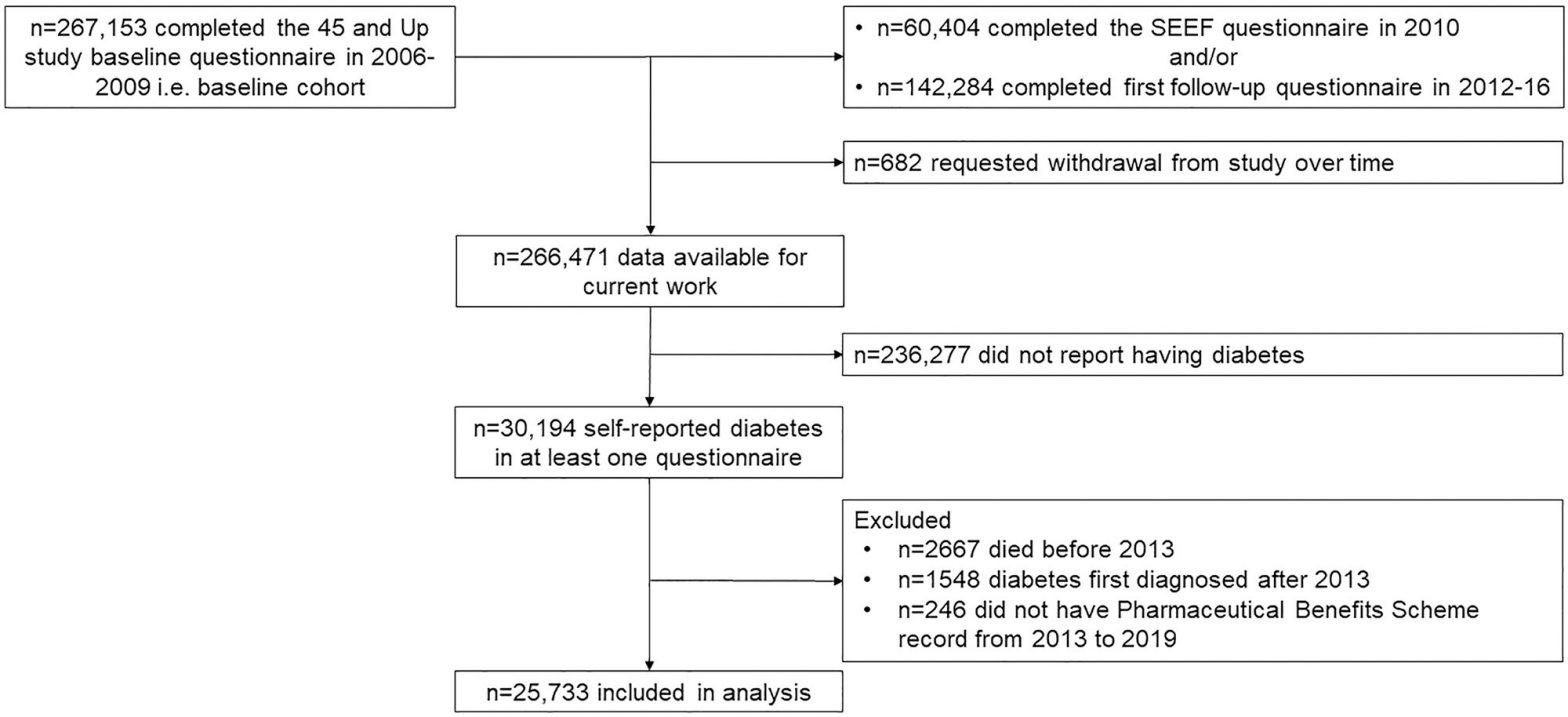
Participant flow diagram.

This study comprised individuals with diabetes participating in the Sax Institute’s 45 and Up Study, an ongoing health study [16] that recruited men and women aged ≥45 years from the general population of the Australian state of New South Wales (NSW) randomly sampled from Services Australia (formerly the Australian Government Department of Human Services) Medicare Australia enrolment database. In brief, Medicare is the universal health insurance scheme in Australia, which allows Australian citizens and permanent residents, New Zealand citizens, and visitors from countries with reciprocal health care agreements, living in Australia to access free or subsidised health services. NSW residents aged ≥45 years were randomly sampled with oversampling in people aged ≥80 years and those who lived in rural and remote areas. Altogether, 267,153 individuals completed a self-administered baseline questionnaire in 2006–2009 and consented to follow-up and linkage of their information to health administrative databases. In 2010, 60,404 of the first 100,000 enrolled participants completed the Social, Economic and Environmental Factors Study (SEEF) questionnaire. In 2012–2016, 142,284 of all participants completed the first follow-up questionnaire. Since participants can request to withdraw from the Study at any time, the size of the available cohort will vary. For this project, data from 266,471 participants were provided (Fig 1).

The Centre for Health Record Linkage used probabilistic matching to link questionnaires of participants with their hospital and mortality records in the NSW Admitted Patient Data Collection and Registry of Birth, Deaths and Marriages, where records with an uncertain probability of being true matches were checked by hand (http://www.cherel.org.au). PBS/RPBS records of participants, supplied by Services Australia, were linked using deterministic matching. Participants were included for analysis if they answered “Yes” to “Has a doctor ever told you that you have diabetes?”; had diabetes diagnosed before 2013 based on their reported age when diabetes was first found; were alive on 1^st^ January 2013; and had at least one PBS medication record from the period 1^st^ January 2013 through 31^st^ December 2019. Therefore, this study included 25,733 participants who self-reported having diabetes in at least one questionnaire after excluding 4461 participants who died before 2013, were diagnosed with diabetes after 2012, or who had no PBS records from 2013 onwards (Fig 1).

All participants provided written consent through the return of consent form completed along with the baseline questionnaire. The 45 and Up Study was approved by the University of NSW Human Research Ethics Committee and the use of linked data for this project was approved by the NSW Population and Health Services Research Ethics Committee.

### Medication dispensing data

PBS subsidised glucose lowering medications (Anatomical Therapeutic Chemical classification code [17]: A10), lipid modifying agents (C10), and blood pressure lowering medications (C02–C04, C07–C09) were assessed. Analyses were restricted to PBS data collected from 1^st^ January 2013 onwards as all subsidised medications for both concessional and general recipients have been collected since mid-2012 [18].

### Statistical analysis

Medication use was reported for each calendar year from 2013 through 2019 by sex and age group in 2013. Age in 2013 was used because baseline data were collected over a four-year period and medication use was determined using PBS data from 2013 onwards. The percentage of participants who were supplied with specific drug classes in each year under study was calculated as the number of participants with at least one dispensing record of the drug class within the calendar year divided by the number of participants alive on 1^st^ January of that year. Percentages were reported for any glucose lowering medications, specific glucose lowering medications drug classes (ie. metformin, sulfonylureas, dipeptidyl peptidase-4 inhibitors (DPP-4i), thiazolidinedione, acarbose, insulin, glucagon-like peptide 1 receptor agonists (GLP-1 RA) and sodium glucose co-transporter 2 inhibitors (SGLT2i)), any lipid modifying agents, and any blood pressure lowering medications.

The number of drug classes used concurrently for glucose lowering medications, lipid modifying agents, and blood pressure lowering medications, respectively, were estimated as the median of the total number of drug classes supplied concurrently over a calendar year, where the period of medication covered was calculated as the supply date plus the standard coverage days for specific glucose lowering medication drug classes [9] or the date of supply plus the PBS maximum quantity unit for specific lipid modifying agents or blood pressure lowering medications in the PBS dataset. For medications resupplied before the end date of the previous supply, the coverage period was calculated as the end date for the previous supply plus the standard coverage days or PBS maximum quantity unit. For insulin users, the percentage who used insulin as monotherapy, dual therapy, triple therapy, or multiple therapy were also reported by sex and age groups.

For each glucose lowering drug class, we used logistic regression to estimate the age and duration of diabetes adjusted percentages of participants who were supplied with the drug class by sex and body weight status (normal weight, overweight, obesity class 1, and obesity class 2 and above) at baseline by year of medication supply from 2013 to 2019. For GLP-1 RA and SGLT2i, we also estimated the annual sex-specific percentage use by history of cardiovascular disease, residential remoteness, and socioeconomic status of residence. Participants with a hospital principal diagnosis of myocardial infarction, stroke and/or heart failure (classified according to the tenth revision of the International Classification of Disease: I21-I23, I50, I60-I61, I63-I64) and/or stenting as a principal procedure (classified according to the Australian Classification of Health Interventions) before 1^st^ January 2013 were considered to have a history of cardiovascular disease. The Socio-Economic Indexes for Areas–Index of Relative Socio-Economic Disadvantage [19] was used to assign five socioeconomic status groups. The annual sex-specific percentages were adjusted for age, duration of diabetes and body mass index (BMI). Sex differences in participant characteristics were tested using t-tests for means and chi-square tests for proportions. Adjusted sex differences between subgroups were evaluated using logistic regression. All statistical analyses were performed using Stata/MP V.16.0.

## Results

Of the 25,733 participants who met the inclusion criteria, 45.2% were women (Table 1). Women were more likely than men to be in younger age groups; to live in outer regional/remote/very remote areas and in the most socioeconomically disadvantaged areas; to have a shorter duration of diabetes, obesity class 2 and above, lower income and lower educational attainment; but were less likely to have a history of cardiovascular disease.

**Table 1:** Characteristics of participants with self-reported diabetes in the 45 and Up Study.

Socio-demographic characteristics^a^	Men	Women	p-value
**N (%)**	14,108 (54.8)	11,625 (45.2)	
**Mean age (SD)**	71.2 (9.8)	70.1 (10.6)	<0.001
**Age group (years)**			
45–54	3.8%	6.6%	<0.001
55–64	23.2%	26.6%	<0.001
65–74	36.1%	32.9%	<0.001
≥75	36.9%	34.0%	<0.001
**Mean age at diabetes diagnosis (SD)**	57.3 (12.3)	56.2 (13.5)	<0.001
**Duration of diabetes (years)**			
0–2	4.8%	5.6%	0.006
3–9	34.5%	36.4%	0.002
10–19	41.6%	38.7%	<0.001
≥20	19.2%	19.4%	0.73
**Mean body mass index (SD)**	29.4 (5.0)	30.4 (6.3)	<0.001
**Weight status**			
Normal weight (BMI 18.5–24.9 kg/m^2^)	17.6%	19.9%	<0.001
Overweight (BMI 25.0–29.9 kg/m^2^)	42.6%	31.9%	<0.001
Obesity class 1 (BMI 30.0–34.9 kg/m^2^)	27.1%	26.3%	0.17
Obesity class 2 and above (BMI ≥35.0 kg/m^2^)	12.7%	21.9%	<0.001
**Regular smoker**	7.3%	7.0%	0.35
**Area of residence**			
Major cities	54.1%	52.2%	0.003
Inner regional	34.9%	35.1%	0.68
Outer regional/remote/very remote	11.1%	12.7%	<0.001
**Socio-Economic Indexes for Areas Index of Relative Socio-economic Disadvantage**			
Most disadvantaged fifth	25.8%	29.9%	<0.001
Second fifth	23.1%	23.7%	0.29
Third fifth	18.9%	18.3%	0.24
Fourth fifth	15.9%	15.0%	0.045
Least disadvantaged fifth	16.2%	13.1%	<0.001
**Education**			
No school certificate or other qualification	15.2%	20.2%	<0.001
School/higher school certificate	27.6%	41.9%	<0.001
Trade/apprenticeship/diploma	37.7%	23.7%	<0.001
University degree or higher	19.5%	14.2%	<0.001
**Income (per year)**			
<$30,000	41.3%	45.1%	<0.001
$30,000–69,999	26.2%	20.3%	<0.001
≥$70,000	18.7%	11.4%	<0.001
Rather not say	13.8%	23.1%	<0.001
**Hospital recorded history of cardiovascular disease before 2013**	54.8%	48.9%	<0.001

^a^ All socio-demographic factors measured at baseline except for age, duration of diabetes and history of cardiovascular disease determined in 2013

BMI = body mass index.

### Use of glucose lowering medications

**Fig 2:**
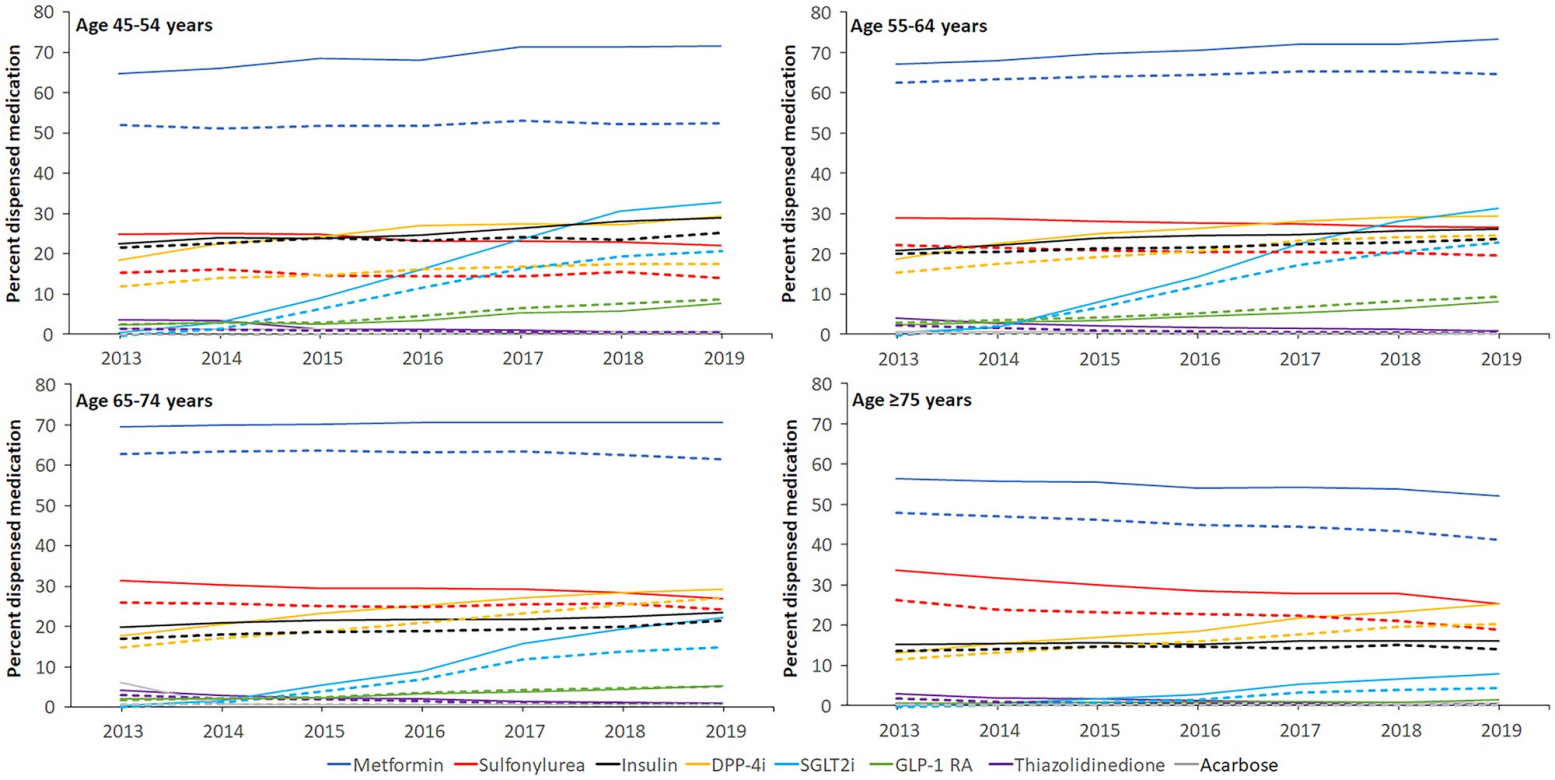
Percentages of participants supplied with glucose lowering medications by drug class, sex and age. Based on age in 2013. Solid line: Men, Dashed line: Women. DPP-4i = dipeptidyl peptidase-4 inhibitor; SGLT2i = sodium-glucose cotransporter-2 inhibitor; GLP-1 RA = glucagon-like peptide-1 receptor agonist.

The percentages of individuals treated every year with glucose lowering medications are reported in Table 2. The percentage of women who were supplied with any glucose lowering medication at least once during a calendar year was consistently lower than men over the seven-year period. Across all age-groups and every calendar year, the annual percentage of men supplied with a glucose lowering medication was ≥7.1 percentage points higher than that of women whereas within age-groups, it was ≥4.9 percentage points higher than that of women. Among men, in every calendar year the annual percentage of those supplied with any glucose lowering medication in the ≥75 years age-group were 3.1–15.4 percentage points lower than the 45–54 age-group, 5.0–15.6 percentage points lower than the 55–64 age-group, and 5.7–13.1 percentage points lower than the 65–74 age-group. Among women, the percentage was lower in those aged ≥75 years compared to 55–64 and 65–74 age groups over the seven-year period, and those aged 45–54 years in 2018 and 2019 (p<0.03 for all comparisons). In both women and men, the percentage of participants aged ≥75 years who were supplied with any glucose lowering medication decreased over time, while the percentage in the younger age groups increased or remained relatively unchanged over the same period. In all sex and age groups, metformin was the most commonly supplied drug and acarbose the least (Fig 2). Since the PBS listing of SGLT2i in 2013, the percentage of participants supplied with SGLT2i increased to 33% and 31% in men 45–54 and 55–64 years, respectively, in 2019, similar to 2019 rates for sulfonylureas, DPP-4i and insulin in these age groups. In women, the respective figures were 21% and 23%.

**Table 2:** Percentage of participants supplied with glucose lowering medications by sex and age.

Sex	Year of medication supplied	Age in 2013 (years)				All
		45–54	55–64	65–74	≥75	
Men	2013	77.6%	79.5%	80.2%	74.5%	77.8%
	2014	80.4%	80.3%	81.0%	73.5%	78.1%
	2015	81.2%	81.4%	82.0%	73.1%	78.8%
	2016	80.2%	82.2%	82.3%	71.4%	78.6%
	2017	84.0%	83.4%	82.5%	71.3%	79.2%
	2018	84.9%	84.0%	82.4%	70.8%	79.5%
	2019	85.0%	85.2%	82.7%	69.6%	79.8%
Women	2013	63.1%	73.2%	74.8%	65.4%	70.4%
	2014	63.4%	74.3%	75.8%	64.4%	70.8%
	2015	64.9%	75.3%	76.5%	63.9%	71.4%
	2016	65.1%	75.8%	76.4%	62.8%	71.2%
	2017	66.2%	76.9%	77.6%	62.6%	72.1%
	2018	66.1%	76.9%	77.5%	61.6%	72.0%
	2019	65.7%	77.6%	76.9%	58.9%	71.4%

**Fig 3:**
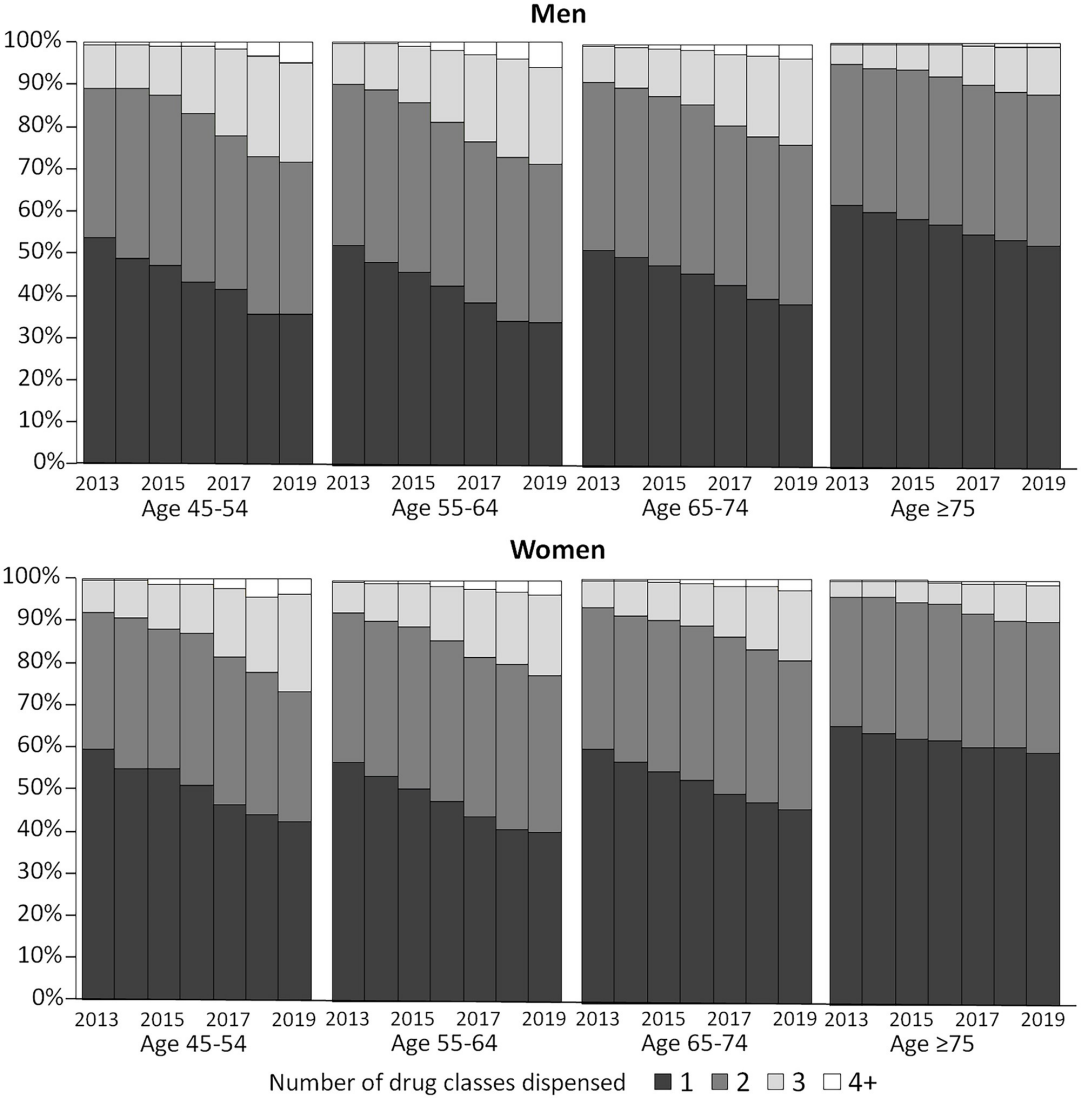
Number of glucose lowering medication drug classes used concurrently by sex and age in 2013 in participants supplied with glucose lowering medications at least once within a calendar year.

The number of drug classes used concurrently increased over time in all sex and age groups with a higher percentage of participants supplied with >1 drug class concurrently in younger age groups (Fig 3). A higher percentage of men than women in all age groups used >1 drug class.

Altogether, 7296 participants were supplied with insulin at least once since 2013. The percentage of participants using insulin monotherapy decreased from 50.1% to 33.2% in men and 52.4% to 36.1% in women between 2013 and 2019 (S1 Table in S1 File). Over the same period, the use of insulin with ≥2 other diabetes therapies increased from 10.6% to 31.6% in men and from 8.7% to 28.0% in women. Among participants on insulin monotherapy in 2013 and alive by the end of the study period, the use of ≥1 diabetes therapy in addition to insulin increased to 13.7% for men and 14.6% for women in 2019.

After excluding 2338 participants who were underweight or missing BMI information, data for 23,395 participants were analysed by weight status. The pattern of drug classes supplied over time was similar between body weight groups. Notable differences included higher percentage of participants who were supplied with SGLT2i and GLP-1 RA in the obesity groups observed in both sexes (S1 Fig in S1 File).

The only statistically significant difference observed in analyses of GLP-1 RA use by history of cardiovascular disease, residential remoteness, and socioeconomic status, was that of use by socioeconomic status (p = 0.003; S2-S4 Tables in S1 File). For SGLT2i use, all differences were small and not statistically significant (p>0.31 for all comparisons).

### Use of lipid modifying agents

The percentage of women who were supplied with any lipid modifying agent at least once during a calendar year was consistently lower than men (p<0.001 for all comparison across calendar years) and, in both sexes, the percentage of participants aged ≥75 years who were supplied with lipid modifying agents decreased over time (p<0.001; Table 3). In participants aged <75 years, the percentage of men and women who were supplied with lipid modifying agents increased over time with higher percentages observed in older age groups. Lower percentages of women aged 45–54 years were supplied with lipid modifying agents than any other age-sex subgroups (p<0.001).

**Table 3:** Percentage of participants supplied with lipid modifying agents by sex and age.

Sex	Year of medication supplied	Age in 2013 (years)				All
		45–54	55–64	65–74	≥75	
Men	2013	62.7%	69.0%	78.6%	78.0%	75.5%
	2014	63.4%	69.3%	79.5%	76.8%	75.6%
	2015	64.7%	71.8%	80.2%	76.3%	76.2%
	2016	65.7%	72.5%	80.8%	74.8%	76.1%
	2017	69.2%	75.1%	80.6%	73.8%	76.6%
	2018	71.1%	75.9%	81.3%	70.7%	76.2%
	2019	72.5%	77.3%	81.3%	68.9%	76.4%
Women	2013	45.8%	66.8%	77.7%	75.5%	72.0%
	2014	47.4%	66.8%	77.5%	73.4%	71.2%
	2015	49.5%	68.7%	78.3%	71.6%	71.6%
	2016	51.2%	69.7%	78.2%	69.3%	71.2%
	2017	53.9%	71.5%	79.2%	68.5%	72.0%
	2018	55.2%	72.3%	79.4%	66.9%	72.0%
	2019	53.9%	72.2%	79.4%	64.7%	71.3%

The number of lipid modifying drug classes used concurrently increased over time (S2 Fig in S1 File). Most participants supplied with lipid modifying agents used only one drug class.

### Use of blood pressure lowering medications

The percentage of women who were supplied with any blood pressure lowering medication at least once during a calendar year was lower than for men, but this difference was smaller than that for glucose lowering medication (in 2019: the annual percentage of participants who were dispensed with a blood pressure lowering medication was 2.4 percentage points lower in women than men compared to 8.4 percentage point lower in women than men for glucose lowering medication; Table 4). The percentage of participants aged ≥75 years who were supplied with blood pressure lowering medications decreased over time. Similarly, lower percentages of women aged 45–54 years were dispensed with blood pressure lowering medications compared to other age-sex groups (p<0.001).

**Table 4:** Percentage of participants supplied with blood pressure lowering medications by sex and age.

Sex	Year of medication supplied	Age in 2013 (years)				All
		45–54	55–64	65–74	≥75	
Men	2013	60.9%	71.7%	83.1%	84.5%	80.1%
	2014	64.4%	72.9%	83.5%	83.9%	80.4%
	2015	66.4%	74.6%	83.8%	83.3%	80.7%
	2016	66.5%	75.5%	84.5%	81.4%	80.5%
	2017	67.3%	76.4%	84.4%	80.0%	80.2%
	2018	69.0%	77.0%	84.1%	78.6%	79.9%
	2019	68.0%	78.3%	83.2%	77.2%	79.5%
Women	2013	48.9%	68.6%	81.2%	86.7%	77.6%
	2014	51.0%	69.6%	81.4%	86.1%	77.7%
	2015	51.6%	70.3%	81.8%	85.2%	77.7%
	2016	52.8%	71.3%	82.5%	84.6%	78.0%
	2017	53.8%	72.3%	82.6%	84.0%	78.0%
	2018	56.3%	73.0%	82.7%	82.4%	77.8%
	2019	56.9%	72.6%	82.2%	80.9%	77.1%

The percentage of participants who were supplied with >1 drug class concurrently among users was lowest in those aged 45–54 years (S3 Fig in S1 File). In those aged ≥75 years, the percentage who were supplied with ≥4 drug classes concurrently decreased over time.

## Discussion

In this large Australian diabetes cohort, the usage of glucose lowering medications, lipid modifying agents, and blood pressure lowering medications was relatively high [7]. Nevertheless, women were less likely to fill these medications than men of similar age, with the largest discrepancy for glucose lowering medications. While the overall sex-specific percentage of medication use remained relatively unchanged between 2013 and 2019, it decreased in participants aged ≥75 years. The use of insulin with ≥2 other diabetes therapies increased substantially between 2013 and 2019 in both sexes. Despite the acknowledged benefit of GLP-1 RA and SGLT2i [20–22], use of these drug classes was not more common in participants with a history of cardiovascular disease.

Better glucose control and cardiovascular risk factor management in our cohort of women, especially those in the youngest age group, could be a reason for the lower percentages of medication filled compared to men. As biomedical assessment was not conducted in the 45 and Up Study, we were unable to determine the health status of participants. However, better management is unlikely the reason as our cohort of women had lower educational attainment and were more likely to live in the most disadvantaged areas compared to men and are likely to have had higher rates of risk factors and chronic conditions as reported for lower socioeconomic groups in Australia [23]. This observation coupled with our findings of medication dispensing rates suggests potential sex disparities in the management of people with diabetes.

Medication use in the oldest age group declined over time while the percentage of those who used >1 drug class concurrently increased for glucose lowering medications. The addition of drug classes over time is likely the result of participants not achieving HbA1c target with existing pharmacotherapy with the Australian type 2 diabetes management algorithm recommending adding second- and third-line agents when HbA1c target is not reached [24]. The decline in medication use in the oldest age group is consistent with current diabetes management in Australia with less intensive treatment in older people recommended to reduce the risk of hypoglycaemia and minimise the use of multiple medications [25].

Differences in the pattern of other glucose lowering medication prescribing with insulin in people with type 1 and type 2 diabetes could not be accurately assessed because the Study did not collect information on diabetes type. People who used insulin for the first time after 2013 most likely had type 2 diabetes. The use of insulin in this group increased substantially from 15% of men and 12% of women in 2014 to 37% of men and 36% of women in 2019. Conversely, some participants who used insulin only in 2013 and subsequently had other medications added, may have had type 1 diabetes.

The higher percentage of GLP-1 RA and SGLT2i use in the groups with obesity after accounting for age and duration of diabetes suggests that doctors are aware of the weight loss benefit of these medications. However, we did not see a wider use in participants with a history of cardiovascular disease. Our results were consistent with the management guidelines in Australia during the study period, which stated the weight loss benefit but not the cardiovascular benefits of these two drug classes [26]. Moreover, sulfonylurea and DPP-4i were recommended as second- and third-line treatment options over SGLT2i and GLP-1 RA at that time. A US study of people with pharmacologically treated diabetes reported people with history of myocardial infarction, heart failure and kidney disease were less likely to start SGLT2i [27]. It has been suggested that the low prescribing rate of SGLT2i in people with diabetes and cardiovascular disease could be due to doctors being unfamiliar with using this drug [28]. A recent Australian study which interviewed general practitioners in NSW identified barriers to prescribing SGLT2i included low awareness of its cardiovascular effect and patients’ encounter of adverse effects from SGLT2i use [29]. Furthermore, contrasting to a study which reported individuals who resided in remote areas were less likely than those in major cities to receive GLP-1 RA and SGLT2i in the first years since these drug classes were listed in the PBS [30], our results did not suggest an association between filling of GLP-1 RA or SGLT2i and area of residence. With the current guidelines recommending SGLT2i, or GLP-1 RA if intolerance or contraindication to SGLT2i, as an addition to other glucose lowering medications in people with diabetes and cardiovascular disease, multiple cardiovascular risk factors and/or kidney disease [31], uptake of SGLT2i and GLP-1 RA for people with diabetes and at high cardiovascular risk should improve in clinical practice.

The strength of this study was the use of the full PBS/RPBS dataset in combination with socio-demographic information of participants from the 45 and Up Study questionnaires, medical history from hospital records and mortality status from death records. Nevertheless, self-reported diabetes was used to define the cohort and information on diabetes type was not collected. The Study was not designed to be representative of the Australian general population [32], however, there is no reason to assume medication use in people with diabetes differs between Australian jurisdictions. Furthermore, we were unable to determine the extent of primary medication non-adherence (i.e. not getting or delay getting prescribed medication) and whether it was more prevalent in certain subgroups. With the lack of biomedical data, we were unable to determine the respective proportions of participants who achieved treatment targets in those who used medications for glucose lowering, blood pressure lowering, and/or lipid modifying.

The apparent underuse of glucose lowering medications and cardiovascular medications in women with diabetes in Australia needs further investigation. Awareness of potential sex disparities and inequalities in diabetes management need to be addressed through increasing awareness among health professionals. SGLT2i and GLP-1 RA could be prescribed more widely to individuals with diabetes and obesity or high cardiovascular risk in all age groups to take advantage of the weight loss and cardiovascular protective effects.

## Supporting information

S1 File Online supplementary materials. (PDF)
