# Supplementary material for: Sex and age differences in the use of medications for diabetes and cardiovascular risk factors among 25,733 people with diabetes
Source: PLoS One. 2023 Oct 24;18(10):e0287599. doi: 10.1371/journal.pone.0287599 (PMC10597478; doi:10.1371/journal.pone.0287599)

Sex and age differences in the use of medications for diabetes and cardiovascular risk factors among 25,733 people with diabetes

Crystal M.Y. Lee, Alice A. Gibson, Jacob Humphries, Natasha Nassar, Stephen Colagiuri

S1 Table. Number of glucose lowering medication drug classes used concurrently in insulin users by sex and age

Sex	Age in 2013 (years)	Insulin used as	2013	2014	2015	2016	2017	2018	2019
Men	All	N	2603	2638	2648	2578	2532	2510	2475
		Monotherapy (%)	50.1	45.5	44.1	41.0	38.3	34.5	33.2
		Dual therapy (%)	39.3	41.6	40.8	40.0	35.7	35.3	35.1
		Triple therapy (%)	9.8	12.1	12.8	17.1	22.7	25.6	25.9
		Multiple therapy (%)	0.8	0.9	1.3	1.9	3.4	4.7	5.7
		Did not use insulin in 2013 (%)	0	15.1	20.3	24.8	29.1	33.6	37.1
	Insulin only users[a]	N	556	556	556	556	556	556	556
		Monotherapy (%)	100	93.5	89.7	83.1	81.1	77.9	75.5
		Dual therapy (%)	0	2.5	4.5	8.1	9.2	10.6	10.6
		Triple therapy (%)	0	0	0.5	0.4	2.0	3.1	3.1
		Multiple therapy (%)	0	0	0	0	0.2	0.2	0
		No dispensing record (%)	0	4.0	5.2	8.5	7.6	8.3	10.8
	45–54	N	120	128	126	128	136	144	147
		Monotherapy (%)	56.7	53.9	49.2	48.4	47.1	38.2	39.5
		Dual therapy (%)	30.0	33.6	38.9	32.0	27.9	31.9	29.9
		Triple therapy (%)	10.8	11.7	9.5	17.2	21.3	22.9	23.8
		Multiple therapy (%)	2.5	0.8	2.4	2.3	3.7	6.9	6.8
	55–64	N	682	719	765	776	773	796	798
		Monotherapy (%)	47.9	42.7	40.4	37.2	34.8	30.9	30.7
		Dual therapy (%)	39.1	42.7	40.4	39.0	32.5	30.5	30.3
		Triple therapy (%)	12.3	13.9	17.5	21.4	28.6	32.0	29.7
		Multiple therapy (%)	0.6	0.7	1.7	2.3	4.1	6.5	9.3
	65–74	N	1004	1039	1044	1035	1008	1012	1029

		Monotherapy (%)	44.0	38.7	38.7	35.7	32.8	29.8	28.4
		Dual therapy (%)	44.3	46.3	44.1	44.0	38.7	38.8	39.4
		Triple therapy (%)	10.6	13.6	15.9	18.0	24.2	26.7	27.3
		Multiple therapy (%)	1.1	1.4	1.3	2.3	4.3	4.6	5.0
	≥75	N	797	752	713	639	615	558	501
		Monotherapy (%)	58.6	56.0	55.1	52.4	49.6	47.0	45.3
		Dual therapy (%)	34.5	35.5	36.9	36.5	36.4	36.4	35.5
		Triple therapy (%)	6.4	8.2	7.4	10.3	13.2	15.1	17.8
		Multiple therapy (%)	0.5	0.3	0.6	0.8	0.8	1.6	1.4
Women	All	N	1995	2026	2080	2037	2017	2026	2035
		Monotherapy (%)	52.4	47.8	45.3	45.1	40.4	38.3	36.1
		Dual therapy (%)	38.9	40.9	41.3	38.6	38.2	37.1	35.9
		Triple therapy (%)	8.4	10.7	12.2	14.5	18.5	21.2	23.4
		Multiple therapy (%)	0.3	0.6	1.2	1.8	2.9	3.5	4.6
		Did not use insulin in 2013 (%)	0	12.4	19.2	22.8	27.7	31.9	36.4
	Insulin only users[a]	N	494	494	494	494	494	494	494
		Monotherapy (%)	100	93.3	90.9	85.6	80.4	78.7	75.7
		Dual therapy (%)	0	3.2	4.9	5.9	9.1	10.3	13.2
		Triple therapy (%)	0	0.2	0.2	1.4	2.0	2.4	1.2
		Multiple therapy (%)	0	0	0	0	0.2	0.2	0.2
		No dispensing record (%)	0	3.2	4.0	7.1	8.3	8.3	9.7
	45–54	N	167	176	184	178	184	179	192
		Monotherapy (%)	52.1	48.3	44.6	46.1	41.3	40.8	36.5
		Dual therapy (%)	36.5	39.2	38.0	33.1	32.1	34.6	29.2
		Triple therapy (%)	10.8	11.9	14.1	18.5	23.9	19.6	28.6
		Multiple therapy (%)	0.6	0.6	3.3	2.2	2.7	5.0	5.7
	55–64	N	631	634	663	661	684	690	708
		Monotherapy (%)	47.1	41.2	38.2	36.6	32.7	29.1	29.7
		Dual therapy (%)	41.4	44.6	45.6	40.8	38.5	39.9	36.9
		Triple therapy (%)	11.3	13.2	14.6	20.1	24.6	25.5	27.3
		Multiple therapy (%)	0.3	0.9	1.7	2.4	4.2	5.5	6.2
	65–74	N	645	680	701	696	695	710	752

	Monotherapy (%)	49.9	45.7	42.1	43.8	40.9	38.9	36.0
	Dual therapy (%)	40.9	40.7	43.7	39.7	39.4	35.4	36.2
	Triple therapy (%)	8.8	12.8	13.3	14.4	16.7	23.1	23.5
	Multiple therapy (%)	0.3	0.7	1.0	2.2	3.0	2.7	4.3
≥75	N	553	536	532	502	454	447	383
	Monotherapy (%)	61.5	58.2	58.8	57.6	50.7	50.3	48.0
	Dual therapy (%)	34.4	37.1	34.2	36.1	38.5	36.7	36.8
	Triple therapy (%)	4.0	4.7	7.0	6.0	9.9	12.1	13.6
	Multiple therapy (%)	0.2	0	0	0.4	0.9	0.9	1.6

[a] Participants who used only used insulin in 2013 and were alive on 31 December 2019

S2 Table. Age, duration of diabetes and body mass index adjusted percentages of participants supplied with GLP-2 RA or SGLT2i by sex and hospital recorded history of cardiovascular disease[a]

Drug class	Sex	Hospital event before 2013	N in 2013	2013	2014	2015	2016	2017	2018	2019	p-value	
											CVD group	time
GLP-1 RA	Men	No cardiovascular disease	11925	1.6%	2.0%	2.1%	2.8%	3.4%	3.8%	4.9%	0.70	<0.001
		Cardiovascular disease	2183	1.9%	1.7%	2.0%	3.2%	3.3%	3.7%	5.4%		
	Women	No cardiovascular disease	10646	1.9%	2.2%	2.5%	3.3%	4.2%	4.9%	5.7%	0.96	<0.001
		Cardiovascular disease	979	2.2%	2.8%	2.6%	3.3%	3.8%	4.0%	5.5%		
SGLT2i	Men	No cardiovascular disease	11925	0%	1.2%	5.0%	8.5%	14.3%	17.9%	20.3%	0.31	<0.001
		Cardiovascular disease	2183	0%	1.5%	5.3%	9.7%	17.0%	20.9%	23.4%		
	Women	No cardiovascular disease	10646	0%	1.5%	4.5%	7.7%	11.7%	13.9%	15.1%	0.94	<0.001
		Cardiovascular disease	979	0%	0.9%	3.6%	7.3%	13.9%	15.8%	17.3%		

GLP-1 RA = glucagon-like peptide 1 receptor agonist; SGLT2i = sodium glucose co-transporter 2 inhibitor; CVD = cardiovascular disease

[a] defined as principal diagnosis of myocardial infarction, stroke and heart failure and/or stenting as principal procedure;

S3 Table. Age, duration of diabetes and body mass index adjusted percentages of participants supplied with GLP-2 RA or SGLT2i by sex and remoteness of residence

Drug class	Sex	Remoteness	N in 2013	2013	2014	2015	2016	2017	2018	2019	p-value	
											remoteness	time
GLP-1 RA	Men	Major cities	7504	1.8%	2.2%	2.3%	2.9%	3.2%	3.5%	4.6%	0.21	<0.001
		Inner regional	4839	1.4%	1.6%	1.8%	2.7%	3.4%	3.9%	4.8%		
		Outer regional/remote/very remote	1540	1.7%	1.5%	1.8%	2.6%	3.8%	4.7%	6.2%		
	Women	Major cities	5976	2.3%	2.5%	2.7%	3.3%	4.2%	4.6%	5.3%	0.25	<0.001
		Inner regional	4019	1.5%	2.0%	2.3%	3.3%	4.3%	4.9%	6.1%		
		Outer regional/remote/very remote	1453	1.6%	2.3%	2.8%	3.5%	3.5%	5.3%	5.7%		
SGLT2i	Men	Major cities	7504	0.1%	1.3%	5.1%	9.2%	15.5%	19.4%	21.5%	0.69	<0.001
		Inner regional	4839	0%	1.1%	5.0%	8.0%	12.9%	16.6%	19.0%		
		Outer regional/remote/very remote	1540	0.1%	1.3%	3.9%	8.1%	14.4%	17.3%	20.5%		
	Women	Major cities	5976	0.1%	1.3%	4.3%	8.2%	12.3%	14.5%	15.3%	0.44	<0.001
		Inner regional	4019	0%	1.6%	4.3%	7.2%	11.5%	13.1%	14.5%		
		Outer regional/remote/very remote	1453	0.1%	1.6%	5.3%	6.8%	11.0%	14.2%	16.2%		

GLP-1 RA = glucagon-like peptide 1 receptor agonist; SGLT2i = sodium glucose co-transporter 2 inhibitor;

S4 Table. Age, duration of diabetes and body mass index adjusted percentages of participants supplied with GLP-2 RA or SGLT2i by sex and socioeconomic status

Drug class	Sex	SEIFA-IRSD	N in 2013	2013	2014	2015	2016	2017	2018	2019	p-value	
											SEIFA	time
GLP-1 RA	Men	Most disadvantaged	3558	1.9%	2.0%	1.9%	2.7%	3.1%	3.8%	4.8%	0.003	<0.001
		2nd fifth	3183	1.4%	1.4%	2.0%	3.1%	3.8%	4.2%	5.2%		
		3rd fifth	2597	1.9%	2.1%	2.1%	2.3%	2.7%	2.9%	3.8%		
		4th fifth	2194	1.4%	1.8%	1.7%	2.5%	3.4%	4.1%	5.5%		
		Least disadvantaged	2236	1.7%	2.6%	2.9%	3.7%	4.0%	3.9%	5.3%		
	Women	Most disadvantaged	3402	1.8%	2.1%	2.5%	3.3%	3.9%	4.4%	5.6%	0.32	<0.001
		2nd fifth	2695	1.6%	1.9%	2.5%	3.3%	4.3%	5.2%	5.7%		
		3rd fifth	2079	2.1%	2.6%	2.3%	3.4%	4.9%	5.1%	6.1%		
		4th fifth	1708	2.4%	2.7%	2.8%	3.5%	4.1%	4.8%	5.6%		
		Least disadvantaged	1491	2.4%	2.6%	3.0%	3.3%	3.8%	4.3%	5.3%		
SGLT2i	Men	Most disadvantaged	3558	0%	1.3%	5.2%	9.0%	13.8%	17.7%	20.3%	0.44	<0.001
		2nd fifth	3183	0%	1.4%	4.6%	8.0%	13.2%	17.1%	19.3%		
		3rd fifth	2597	0.1%	1.1%	5.0%	8.3%	15.2%	18.5%	20.5%		
		4th fifth	2194	0.1%	1.0%	4.4%	8.9%	1.4%	16.7%	19.8%		
		Least disadvantaged*	2236	0.1%	1.1%	5.4%	9.1%	17.1%	21.3%	23.2%		
	Women	Most disadvantaged	3402	0%	1.1%	4.2%	7.6%	11.5%	13.6%	15.0%	0.84	<0.001
		2nd fifth	2695	0%	1.6%	4.0%	7.1%	11.6%	13.9%	15.3%		
		3rd fifth	2079	0.1%	2.0%	5.4%	8.2%	12.6%	14.2%	15.9%		

4th fifth	1708	0.1%	1.6%	5.0%	8.6%	12.6%	14.4%	15.8%
Least disadvantaged	1491	0.4%	1.1%	4.0%	7.3%	10.9%	13.7%	13.4%

GLP-1 RA = glucagon-like peptide 1 receptor agonist; SGLT2i = sodium glucose co-transporter 2 inhibitor; SEIFA-IRSD = Socio-Economic Indexes for Areas – Index of Relative Socio-Economic Disadvantage;

S1 Fig. Age and duration of diabetes adjusted percentages of participants supplied with glucose lowering medications by drug class, sex and body weight status at baseline

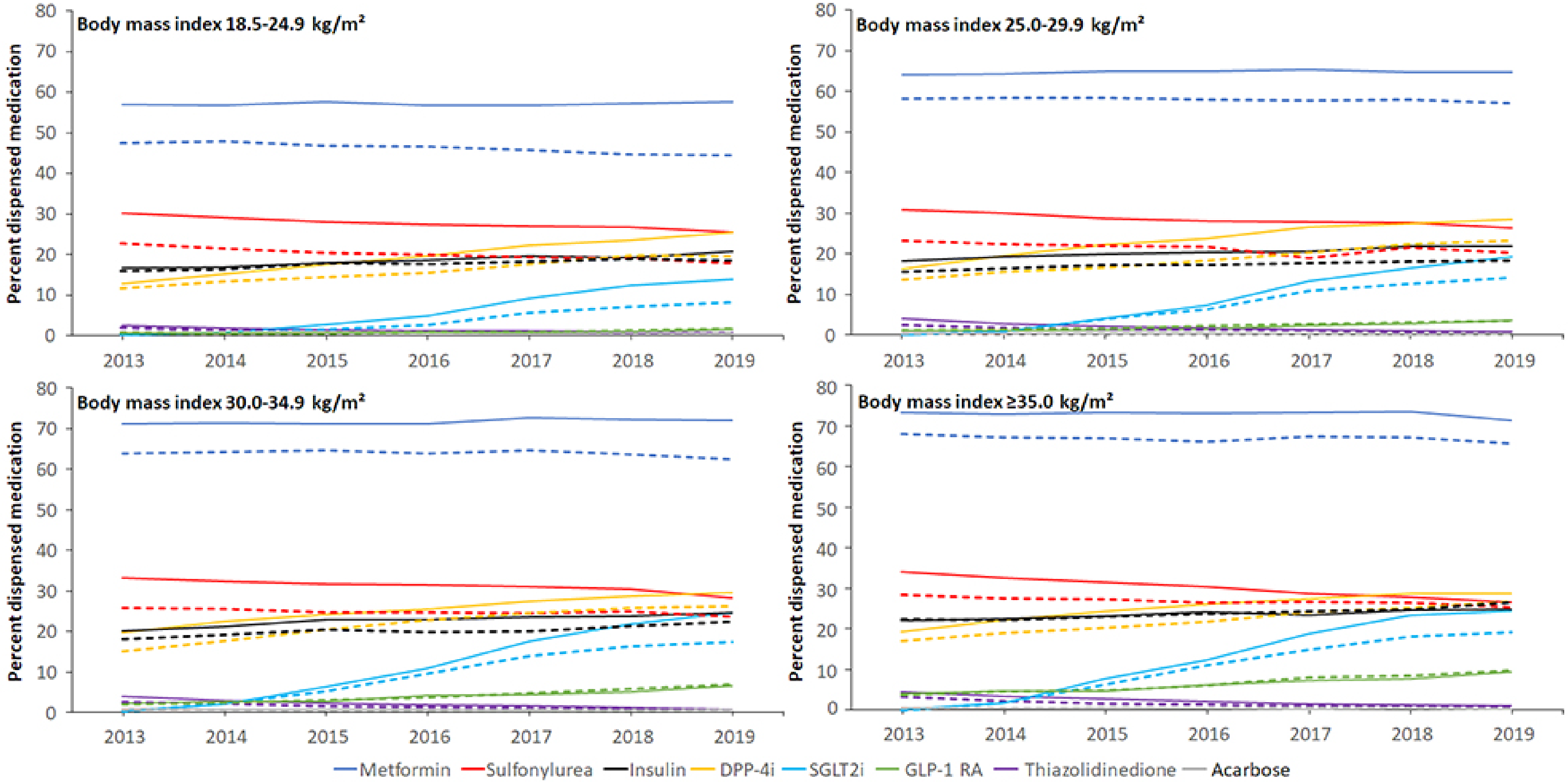

Solid line: Men, Dashed line: Women. DPP-4i = dipeptidyl peptidase-4 inhibitor; SGLT2i = sodium-glucose cotransporter-2 inhibitor; GLP-1 RA = glucagon-like peptide-1 receptor agonist;

S2 Fig. Number of lipid modifying drug classes used concurrently by sex and age in 2013 in participants supplied with lipid modifying agents at least once within a calendar year

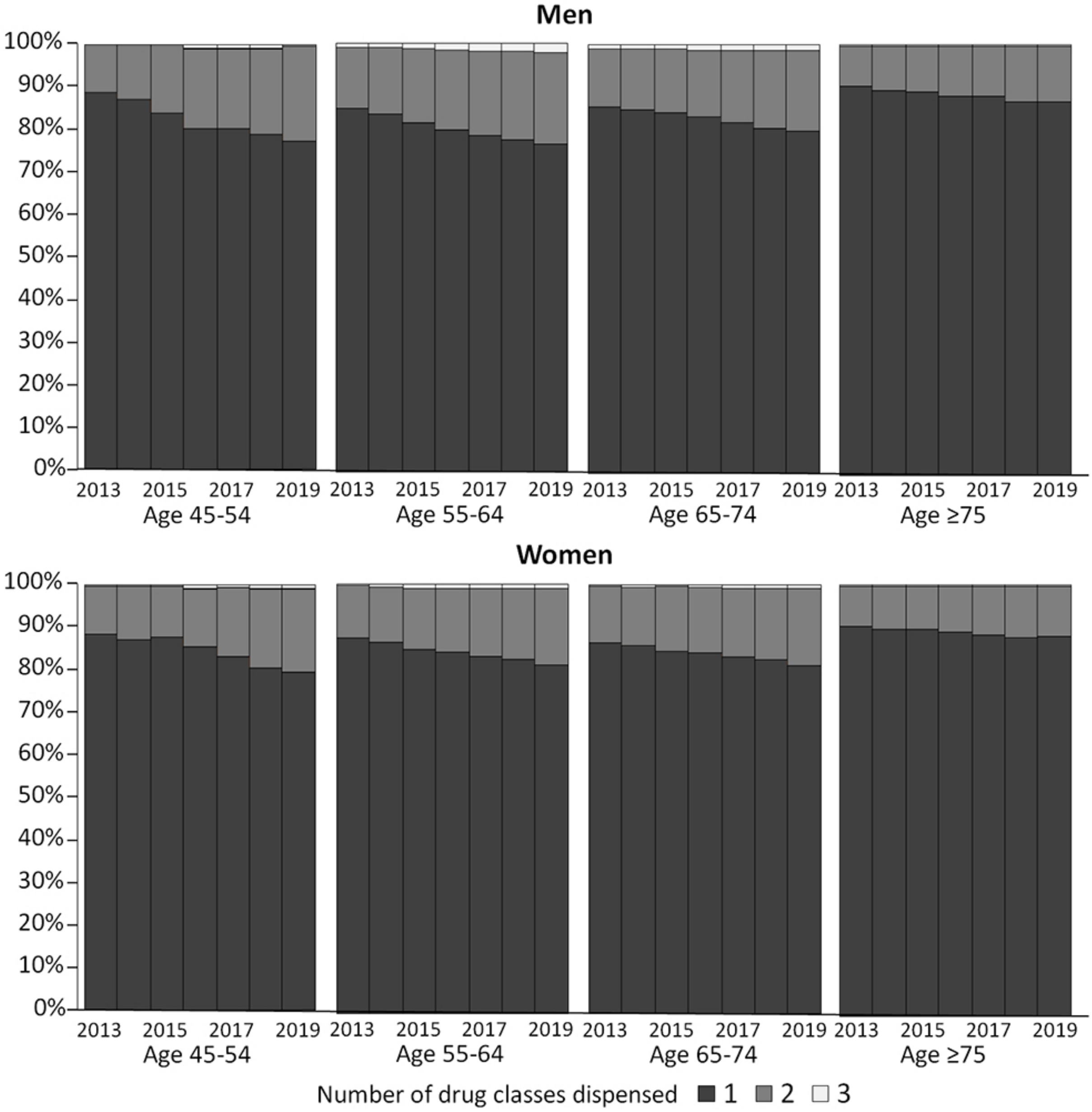

S3 Fig. Number of blood pressure lowering medication drug classes used concurrently by sex and age in 2013 in participants supplied with blood pressure lowering medications at least once within a calendar year

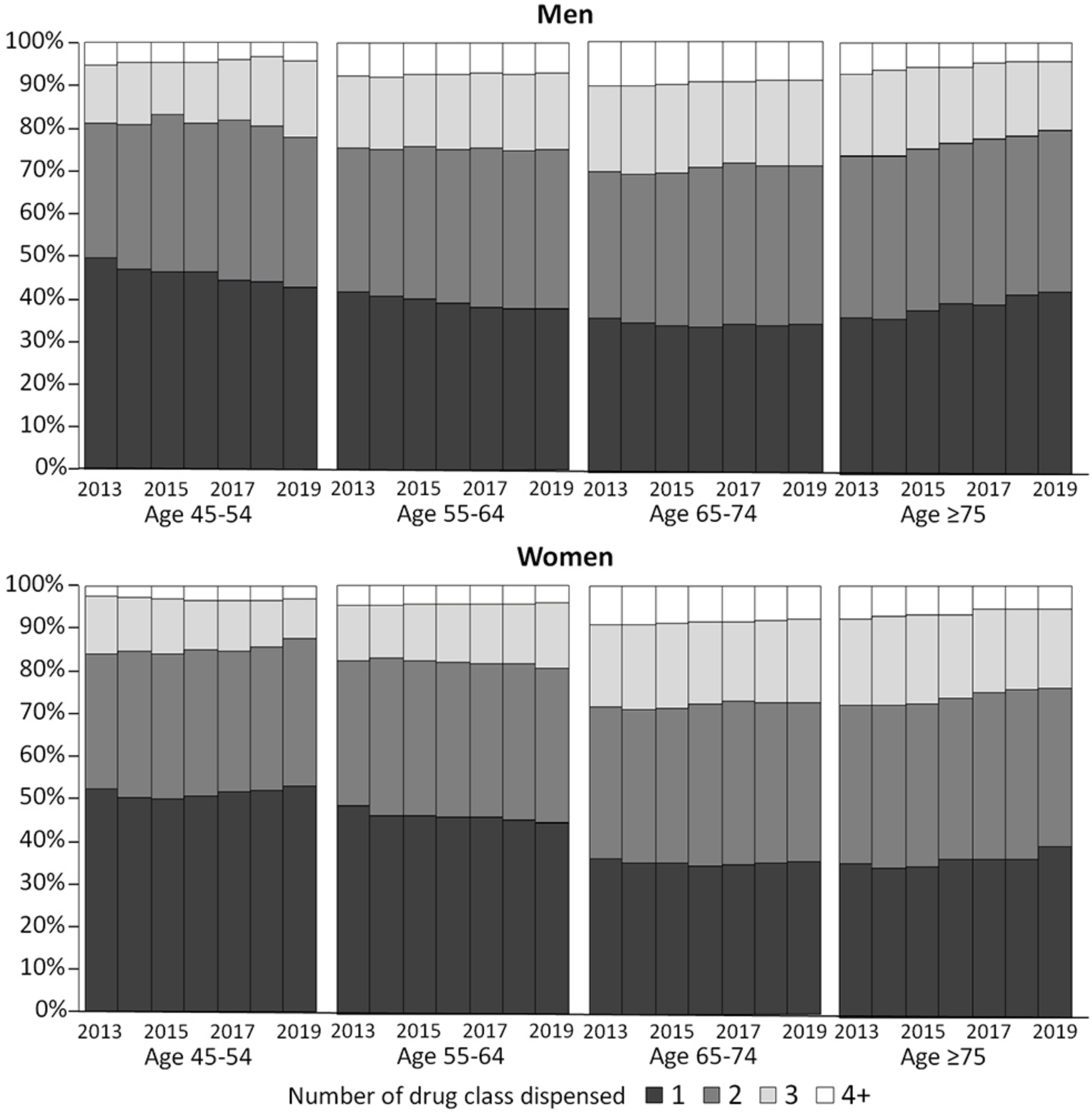

Supplement: S1 File — (PDF) [file pone.0287599.s001.pdf]
